# Trends in CBCT current practice within three UK paediatric dental departments

**DOI:** 10.1007/s40368-020-00526-w

**Published:** 2020-04-24

**Authors:** N. Gallichan, S. Albadri, C. Dixon, K. Jorgenson

**Affiliations:** 1 Liverpool University Dental Hospital, Liverpool, UK; 2 University Dental Hospital of Manchester, Manchester, UK; 3 Newcastle Dental Hospital, Newcastle upon Tyne, UK

**Keywords:** Cone beam computed tomography (CBCT), Paediatric, Clinical indication, Dental radiology

## Abstract

**Introduction:**

Cone beam computed tomography (CBCT) is used across all dental specialties and has a number of advantages compared to 2D images. The SEDENTEXCT guidelines provide a number of indications for the use, however there are currently no specific guidelines for paediatric dentistry. The aim of this study was to assess current practice of CBCT imaging within paediatric dental departments in England, audit compliance of CBCT justifications against the standards set by SEDENTEXCT and assess whether the use of CBCT affected the treatment plan for each individual patient.

**Methods:**

From the retrospective analysis of CBCT examinations taken over a 4-year period across three dental hospitals in the north of England, the following data were collected: age at the time of exposure, clinical indication, region of interest (ROI) and diagnostic findings. Clinical notes were also used to identify whether the CBCT had an effect on the final treatment plan.

**Results:**

A total of 335 CBCT examinations were performed, mean age: 11 years. The number of CBCTs increased each year with a twofold increase in the first 2 years. The main clinical indication in 46% of CBCT examinations was the assessment of localised developing dentition, 68% were in the upper anterior sextant and 61% of CBCT exams were in the mixed dentition age group. The investigations were justified in 100% of the cases.

**Conclusion:**

The quantity of CBCT examination in paediatric dental patients is increasing to assist treatment planning but more often to enable improved surgical planning.

## Introduction

### Cone beam computed tomography (CBCT)

CBCT is a radiographic investigation that creates a three-dimensional image of the exposure site. Developed in the early 1990s, it is increasingly being used in dentistry for various indications. The European Commission has formed the SEDENTEXCT guidelines for radiation protection in CBCT for dental radiology (2012) and a number of other dental specialties have developed specific guidelines (Patel et al. 2014; Isaacson et al. 2015). There are several justifications and clinical indications for the use of CBCT in paediatric dentistry, indicated by *SEDENTEXCT*, which include assessment of the localised developing dentition, assessment of the generalised developing dentition, dental trauma, surgical assessment and endodontic application. Hidalgo-Rivas et al. (2014) found that the most common indication for CBCT examinations in children and young people in United Kingdom (UK) dental hospitals was the localisation of impacted teeth and the detection of root resorption; similarly Van Acker et al. (2016]) found that 36% of the CBCT examinations in their study were prescribed to assess the localized developing dentition. The majority of these examinations were undertaken for orthodontic purpose. Furthermore, Marcu et al. (2018) found the most common justification for CBCT imaging in paediatric patients was for the evaluation of dental anomalies which included monitoring for tooth eruption, treatment planning and orthodontics.

Although CBCT has advantages compared to two-dimensional imaging, the International Commission on Radiological Protection (ICRP) states that the use of CBCT in paediatric patients is of particular concern due to their higher radio sensitivity and smaller size. It generates a higher effective dose in paediatric tissues compared to plain films and has increased stochastic biological effects compared to adults (Aps 2013). It is therefore imperative that as paediatric dentists, a CBCT investigation is clearly justified with careful consideration of how it could impact the patients’ treatment, following the principles of IRMER (The Ionising Radiation (Medical Exposure) Regulations (IRMER) No 1322, 2017).

### Clinical indications in paediatric dentistry

Whilst there are European guidelines (SEDENTEXCT) and cumulative literature focused on CBCT use in paediatric patients, there is limited literature as to when and why paediatric dentists require CBCT images. Giray et al. (2019) conducted a cross-sectional questionnaire concluding that about a third of the paediatric dentists who responded had no knowledge of CBCT. Those who did use CBCT imaging, cited their most common reason for use was for pathology of the jaw; whereas Mizban et al. (2019) found the most common reason for a paediatric dentistry consultant to request a CBCT was for unerupted teeth. There are no other studies to our knowledge that investigate the trends of paediatric dentists using CBCT.

The aim of this cross-regional service evaluation was to assess the current practice of CBCT imaging within paediatric dental departments in Northern England. Objectives include:

- Identify the trends of CBCT investigations over four years.
- Compare clinical indications for CBCT requested by paediatric consultants.
- Compare the diagnostic findings from the CBCT examinations.
- Audit compliance of CBCT justifications to the standards set by SEDENTEXCT.
- Assess whether the use of CBCT affected the treatment plan for each individual patient.

## Methods

The project was registered with the relevant clinical effectiveness units and approval was granted. Patient records were anonymised during data collection and patient confidentiality ensured.

A pilot study was conducted which evaluated 3 years of CBCT referral data at hospital site one (H1), and the results demonstrated that CBCTs were increasingly being requested (Gallichan and Albadri 2019). A service evaluation was then conducted by a cross-regional network of paediatric dental departments in Northern England.

In the retrospective analysis of CBCT examinations taken over a four-year period between January 2015 and January 2019, children aged 16 or under were included and the CBCT examinations must have been requested by paediatric dental specialists and not by any other specialty. The age of patient at the time of exposure, clinical indication, region of interest (ROI) and diagnostic findings were identified using digital radiographic programme, Carestream PACS software. Clinical notes were also used to identify whether the CBCT had an effect on the patient treatment plan and any documented description of how it affected the treatment plan was noted and analysed.

The clinical indication for each exam was recorded and grouped using the standards set by SEDENTEXCT (2013): localised developing dentition, generalised developing dentition, dental trauma, endodontics and surgical assessment. ROI was classified in sextants if more than one ROI was requested. Age of the patient was also grouped into one of the three categories, ≤ 6 years, 7–12 years, and 13–16 years. Diagnostic findings were defined as being related to abnormal tooth development, pathology, trauma related, and others including ectopic and failure to erupt. Statistical analysis using IBM SPSS 25 was performed to compare mean age.

## Results

### Demographic data

**Fig. 1:**
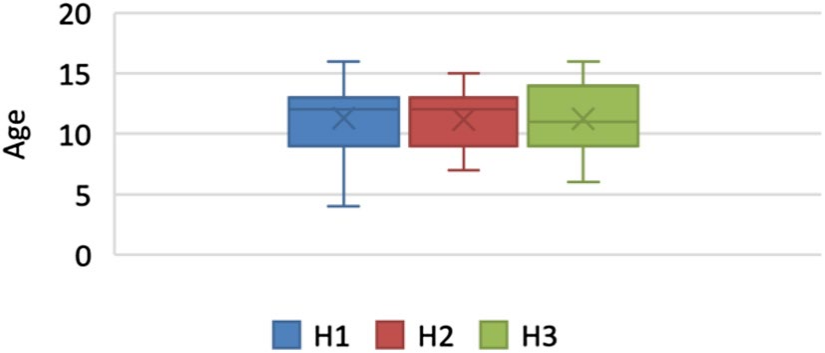
Distribution of age at the time of CBCT exposure, comparison of three dental hospitals

A total of 335 CBCT examinations were requested by paediatric dentists over a four-year period. This represented 3.7% of all CBCTs taken in the dental hospitals and 27% of the total number of CBCTs requested for children aged 16 or under across the three units. The average age of children was 11 years (SD 2.7 years) with a distribution age range between 4 and 16 years (Fig. 1). There was no significant difference between the mean age in the three hospitals, which were 11.1 ± 2.8 years (H1), 11.1 ± 2.9 years (H2), and 10.8 ± 2.5 (H3). Paediatric dentists in H2 requested the highest number of CBCTs (164), followed by H1 (129) and H3 (*n* = 42). Tables 1 and 2 illustrate the distribution of age groups. Age groups were categorised by the average for dentition type; primary dentition (3–6 years), mixed dentition (7–12 years) and permanent dentition (13–16 years). The majority of patients (204, 61%) were in the mixed dentition age group.

**Table 1:** Indications for CBCT defined by age category

Indication	Age group			Total	(%)
	3–6 years	7–12 years	13–16 years		
Localised developing dentition	6	107	42	155	46
Generalised developing dentition	1	10	2	13	4
Dental trauma	3	23	19	45	13
Surgical assessment	4	42	19	65	19
Endodontics	0	22	35	57	17
Total	14	204	117	335	99

**Table 2:** The regions of interest of the 335 CBCT and age group correlation Region of interest was grouped into sextant, defined as: (1) upper anterior, (2) upper right posterior, (3) upper left posterior, (4) lower anterior, (5) lower right posterior, (6) lower left posterior, (7) more than one sextant

Region of interest	Age group			Total
	3–6 years	7–12 years	13–16 years	
1	7	149	73	229
2	2	3	5	10
3	1	8	8	17
4	1	9	9	19
5	0	5	2	7
6	1	6	7	14
7	2	24	13	39
Total	14	204	117	335

**Fig. 2:**
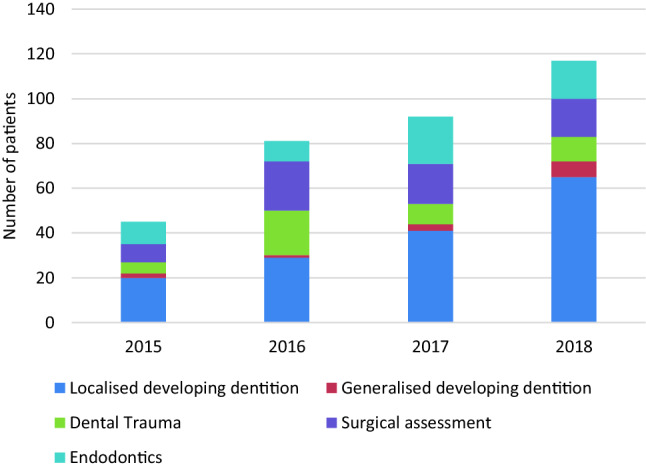
Trends over 4 years for CBCT clinical indications in paediatric patients

The number of CBCT examinations increased each year from 45 in 2015 to 117 in 2018 with a generalised increase each year (Fig. 2). The most significant increase was between 2015 and 2016 when there was an 80% increase from 45 to 81 CBCTs taken across the three dental hospitals.

### Clinical indications

The clinical indications for CBCT investigations were grouped according to the original request into one of the justifications set by SEDENTEXCT (2013). The most common clinical indication for CBCT was consistent, to assess localised developing dentition (155, 46%), Fig. 2. Seventy percent of these CBCTs were taken in the mixed dentition age group (Table 1). The least common clinical indication was to assess the generalised developing dentition; however, it was a prevalent indication across each of the four years and each hospital.

### Region of interest

The majority of the investigations were in the maxilla (256, 76%) with a total of 68% (229) taken in the upper labial sextant. This was the most commonly requested sextant in children aged 7–12 years (73% of ROI) and 13–16 years (62% ROI); in children aged 4–6 years, this sextant represented 50% ROI, as shown in Table 2. More than one sextant was examined in 39 (11%) of the investigations. Within the mandible, 19 CBCTs examined the anterior sextant, 7 posterior right and 14 posterior left, respectively.

### Diagnostic findings

**Fig. 3:**
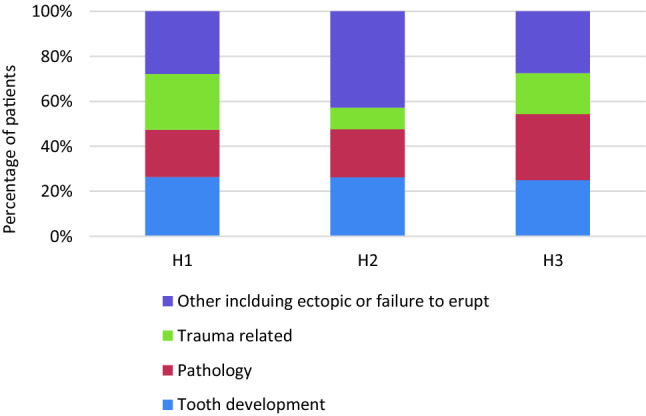
Diagnostic findings from CBCT investigations across three dental hospitals

Diagnostic findings within each CBCT report were categorised into four groups. The most prevalent was an ectopic tooth or supernumery teeth (99, 30%), followed by teeth with abnormal formation or development (86, 26%), and teeth with pathology-associated (84, 25%) and trauma-associated diagnosis (66, 20%). Percentages have been rounded to the nearest whole number. The ratio of each diagnosis at each dental hospital was very similar (Fig. 3).

### Compliance with standards and effect on treatment planning

All CBCT examination requests were deemed to be justifiable according to SEDENTEXCT and therefore complied with the standards. Investigation had a clear documented effect on the treatment planning in 327 cases (97%). There were eight CBCTs where the record-keeping failed to note the CBCT report prior to treatment being implemented, however, from review it was clear that the images had a positive impact on the assisting patient management and treatment.

Descriptions of how CBCT affected the treatment was analysed; the overriding reason was the use of CBCT to aid surgical planning, which was evident across all diagnosis. It was particularly relevant when assessing supplemental teeth, cysts and ectopic teeth. Paediatric dentists found that the CBCT confirmed their diagnosis and analysis of proximity to adjacent local structures, and therefore aided their surgical approach. Analysis also identified how treatment plans were changed because of the use of CBCT. This was most applicable in trauma related cases where the CBCT identified additional findings which had not been clear in 2D images such as resorption, additional fractures, and in some cases, the CBCT rejected those findings which were questioned in 2D images. Finally, CBCTs aided the planning of multidisciplinary cases.

## Discussion

### Trends over 4 years

There are limited publications studying how paediatric dentists use CBCT. This study included CBCTs which were requested by a named consultant in paediatric dentistry only. It is noted that all three dental hospitals involved in this study are based in the North of England and therefore results may not be generalizable. However, these three dental hospitals have a mixed demographic of patients and a mix of academic and hospital staff, and therefore, they are likely to provide a reasonable sample of the UK dental hospitals.

A recent service evaluation at a London-based dental hospital also only included CBCTs requested by paediatric dental specialists and found that the mean age of subjects was 11.5 years and the most common clinical indication for the CBCT examinations was the assessment of unerupted teeth (Mizban et al. 2019). This supports the findings of the present study and echoes the authors’ comment when comparing another UK-based service evaluation (Hidalgo-Rivas et al. 2014) which assessed CBCT in paediatric patients. They found the mean age to be 13.1 years; however, the study was not specific to paediatric dentistry and therefore, the mean age could be higher due to an older cohort of patients requiring CBCT associated with orthodontics. Furthermore, both previous studies included patients aged 17 compared to this study which excluded 17-year-old patients because referrals to the paediatric department are only accepted up to 16 years. This could have affected the mean age in the present study.

One dental hospital in the study had significantly less CBCT investigations requested by paediatric dentists, a total of 42 CBCT examinations. This was due to a staffing shortage of radiologists. Radiology departments are facing increased pressure which can cause long waiting times for patients to have a CBCT examination.

Although there is a clear increasing trend of CBCT use in paediatric dentistry, the ratio of CBCT investigations requested by paediatric dentists is limited compared to other specialties. Only 27% of all CBCTs taken in patients aged 16 and under were requested by paediatric dentists, indicating that the majority of paediatric patients’ CBCTs are taken for orthodontic purposes. In all three units, CBCT requests are vetted by a Dental and Maxillofacial Radiologist to ensure they are justifiable. There are increased radiation risks associated with CBCT in children compared to adults, and the comparison to other specialties and adults highlights how paediatric dentists have remained conservative and vigilant when ordering CBCTs.

### Clinical indications and ROI

The most common indication for requesting a CBCT was to investigate the local developing dentition and when examined within age group categories, this remained consistent in all age groups 4–6, 7–12 and 13–16 years (50%, 73%, 62%) and was succinct with the findings from other studies (Barba et al. 2018; Mizban et al. 2019). The divison of clinical indications was further divided into different groups for these studies whereby in the present study assessment of the localised developing dentiton group included teeth with root resoption unrelated to dental trauma, bony pathology, supernumeries and unerupted teeth.

SEDENTEXCT (2013) list three main justifications for CBCT: developing dentition, restoring the dentition and surgical applications. This study divided developing dention into two subgroups: generalised and localised as well as dividing restoring the dentiton into two subgroups: dental trauma and endodontic uses. The authors recognise that more data could be sought by dividing clinical indications into further localised justifiable subcategories.

A study by Barba et al. (2018) found that within a population sample in San Jose, Costa Rica 100% (*n* = 16) patients < 12 years had a CBCT of the anterior maxilla; however, the most scanned region of interest in adolescents (*n* = 20) was equal in the anterior maxilla, posterior maxilla and posterior mandibular. However, this was a much smaller cohort of paediatric patients compared to both the present study and the study by Hidalgo-Rivas et al. (2014) which found that the most common ROI in paediatric patients is the maxillary canine and incisor region.

This study includes the largest cohort of paediatric patients (< 16 years) referred for CBCT examinations to the best of the authors knowledge. It is also novel in investigating the trends of CBCT investigations taken over a 4-year period, across three UK dental hospitals; providing insight into the use of CBCT in paediatric dentistry.

### Diagnostic findings

This study supports the findings from a (single unit) similar service evaluation by Mizban et al. (2019) who found that CBCT can significantly change the diagnoses and opinions in teeth with dental trauma and pathology associated with developmental anomalies. The impact that CBCT has on treatment planning also supports a statement by IATD (2012) on CBCT: it provides improved visualisation of traumatic dental injuries, particularly root fractures and lateral luxation injuries, monitoring of healing, and complications. In relation to management of cases including root resorption, it was found that CBCT has a positive impact on effecting management, supported by the European Society of Endodontology (Patel et al. 2014).

### Compliance to standards and effect on treatment planning

There is a pattern of similarity in the use of CBCT examinations in paediatric dental patients. All dental hospitals displayed a variety of indications for CBCT and the quantity taken is increasing to assist treatment planning but more often to enable improved surgical planning. All CBCTs taken in 2018 across all dental hospitals had a documented effect on treatment planning, and this could be a result of the disseminated results of previous audit that led to quality improvement. Furthermore, all CBCT requests in the dental hospitals involved are monitored and the prescriptions are assessed by a radiologist. This ensures the units adhere to IRMER (The Ionising Radiation (Medical Exposure) Regulations (IRMER) No 1322, 2017), keeping exposures as low as reasonably possible (ALARP).

Clinical records were analysed retrospectively, however, it was difficult to assess what the original treatment plan or opinion was prior to the CBCT. The records usually raised a question or highlighted two options and the CBCT helped in the decision making. Alqerban et al. (2014) found that the orthodontist had a higher confidence level in the treatment planning of impacted canines when the CBCT was available compared to 2D images. Thematic analysis of the CBCT records identified themes, the most significant theme was to aid surgical planning and to confirm diagnosis. This could suggest that CBCT also increases confidence levels in paediatric dentists in decision making.

This study is based in a dental hospital where access to CBCT can be readily available. However, paediatric dental specialists’ also practice in community dental services and private practices. Further research is necessary to understand the use of CBCT by paediatric dentists in all settings and its effect on their treatment planning. A questionnaire conducted by the members of EAPD and TSPD (Turkish Society of Paediatric Dentistry) reported that 36% of the paediatric dentists had no knowledge of CBCT, and the majority of dentists cited the reason for using CBCT to be for the pathology of the jaws (Giray et al. 2019).

## Conclusion

This study is unique in identifying key trends across several UK dental hospitals. CBCT are increasingly utilised in UK paediatric dental departments, most commonly to assess localised developing dentition. Despite the increase, requests for CBCTs in paediatric dental departments only represent a small percentage of CBCTs in paediatric patients (27%) and in all patients (3.7%). Following the EAPD symposium, May 2019, new guidelines are being created and the results of this project can support these guidelines.
